# The type 1 diabetes candidate genes PTPN2 and BACH2 regulate novel IFN-α-induced crosstalk between the JAK/STAT and MAPKs pathways in human beta cells

**DOI:** 10.21203/rs.3.rs-6079043/v1

**Published:** 2025-03-12

**Authors:** Arturo Roca Rivada, Junior Garcia de Oliveira, Maria Eugenia Martin-Vazquez Garcia, Alexandra Coomans de Brachene, Xiaoyan Yi, Jose Costa Junior, Priscila Zimath, Flore Van Goethem, François Pattou, Julie Kerr-Conte, Antoine Buemi, Nizar Mourad, Décio Eizirik

**Affiliations:** ULB Center for Diabetes Research, Medical Faculty, Université Libre de Bruxelles; ULB Center for Diabetes Research, Medical Faculty, Université Libre de Bruxelles; ULB Center for Diabetes Research, Medical Faculty, Université Libre de Bruxelles; ULB Center for Diabetes Research, Medical Faculty, Université Libre de Bruxelles; Université Libre de Bruxelles; ULB Center for Diabetes Research, Medical Faculty, Université Libre de Bruxelles; ULB Center for Diabetes Research, Medical Faculty, Université Libre de Bruxelles; ULB Center for Diabetes Research, Medical Faculty, Université Libre de Bruxelles; Recherche Translationnelle sur le diabète UMR 1190, Université de Lille, Inserm, Institut Pasteur Lille, CHU Lille; University of Lille, Institut National de la Santé et de la Recherche Médicale (INSERM), Centre Hospitalier Universitaire de Lille (CHU Lille), Institute Pasteur Lille; Pôle de chirurgie expérimentale et transplantation, Institut de recherche expérimentale et clinique, Université catholique de Louvain; Pôle de chirurgie expérimentale et transplantation, Institut de recherche expérimentale et clinique, Université catholique de Louvain; Université Libre de Bruxelles

## Abstract

Type 1 diabetes (T1D) is a chronic autoimmune disease that leads to the progressive loss of pancreatic beta cells. Interferons (IFNs) contribute to the initiation and amplification of beta cell autoimmunity. STAT1 is the main mediator of IFN signalling but little is known on its complex activation processes and role in the progression of beta cell failure. We presently show that two T1D candidate genes (i.e. *PTPN2* and *BACH2*) modulate STAT1 activation via two different pathways, namely the JAK/STAT, involved in the short-term phosphorylation of its tyrosine residue (Y701), and the MAPKs pathway, involved in the long-term phosphorylation of its serine residue (S727). Each STAT1 phosphorylation type can independently induce expression of the chemokine *CXCL10*, but both residues are necessary for the expression of MHC class I molecules. IFN-α-induced STAT1 activation is dynamic and residue-dependent, being STAT1-Y701 fast (detectable after 4h) but transitory (back to basal by 24h) while STAT1-S727 increases slowly (peak at 48h) and is associated with the long-term effects of IFN-α exposure. These pathways can be chemically dissociated in human beta cells by the use of JAK1/2, TYK2 or JNK1 inhibitors. The present findings provide a novel understanding of the dynamics of STAT1 activation and will be useful to develop novel and hopefully targeted (i.e. favouring individuals with particular polymorphisms) therapies for T1D and other autoimmune diseases.

## Introduction

Type 1 diabetes (T1D) is a complex chronic autoimmune disease that leads to progressive loss of pancreatic beta cells. There is no cure for T1D and the standard treatment relies on lifelong exogenous insulin administration^1^. The pathophysiologic mechanisms that trigger T1D are multifactorial and poorly understood^2^. Cytokines, such as interferons (IFNs) and tumor necrosis factor-α (TNF-α), are involved in the innate and adaptative immune responses that contribute to the initiation and amplification of beta cell autoimmunity^1,3,4^. T1D is diagnosed at progressively younger ages, suggesting that the autoimmune process may start early in life^5^, when beta cell and adaptive immune system development and maturation are not yet complete, indicating an important role for the innate immune system and early response cytokines in beta cell failure^6^.

Genome-wide association studies (GWAS) helped to identify more than 80 loci associated with the risk of developing T1D^7^; interestingly, nearly 80% of candidate genes encoded by these loci are expressed in beta cells, pointing toward a direct implication of these cells in the progression and/or the triggering of the disease^2^. A remaining unmet need in the field is to identify the ultimate function of these candidate genes and how to use this knowledge to develop novel and hopefully targeted (i.e. favouring individuals with particular polymorphisms), therapies for the disease. One of the genes identified in these studies is Protein Tyrosine Phosphatase Non-receptor type 2 (*PTPN2*), a tyrosine phosphatase implicated in the control of beta cell physiology, survival, and expansion^8–10^. PTPN2 modulates early immune responses in beta cells, including viral and type I and II IFN responses, protecting the beta cells from excessive cytotoxic signalling in a pro-inflammatory context by directly regulating the JAK/STAT signalling pathway^8,9^. Most known PTPN2 disease-related polymorphisms cause partial loss of function and decreased protein expression^11–13^. Collectively, these observations point to the role of this phosphatase in modulating local islet immune responses and preventing damage caused by excessive inflammation.

Another relevant T1D candidate gene is basic leucine zipper transcription factor 2 (*BACH2*); BACH2 inhibition in human beta cells exacerbates cytokine-induced apoptosis by the mitochondrial pathway of cell death whereas its overexpression has protective effects^4,14^. However, a recent study proposed that BACH2 increases NRF2-dependent antioxidant response genes in mouse models of type 2 diabetes (T2D), preventing beta-cell damage and increasing its function, suggesting that inhibition of BACH2 could be a potential pharmacological intervention in T2D^15^. Of concern, BACH2 is a transcription factor that regulates cytokine-induced PTPN2 expression^14^, and studies based on its inhibition have shown deleterious effects on rodent and human T-regulatory (T-reg) cells^16^. It is thus essential to elucidate the exact signals triggered by BACH2 in beta cells and how its crosstalk with PTPN2 may collaborate to preserve a healthy beta cell in the face of immune-induced stresses.

In a previous study^17^, we showed that the ‘early-response’ cytokines IFN-α and TNF-α have deleterious effects on human beta cells at different stages of development and differentiation, from immature cells (iPSC-derived islet-like cells and the EndoC-βH1 cell model) to fully mature adult cells (human pancreatic islets), and this is aggravated by PTPN2 inhibition. We demonstrated that PTPN2 confers protection against both IFN-α and TNF-α exposure, unveiling an unexpected common downstream signalling pathway between the two cytokines via regulation of the Mitogen-Activated Protein Kinases family (MAPKs) member JNK1^17^.

TNF-α levels have recently been associated with an aggressive form of early-onset T1D^18^ and its inhibition in children and young adults preserves C-peptide production^19,20^. Furthermore, a recent phase-2 clinical study in patients with new-onset T1D showed that the pharmacological inhibition of the Janus kinases (JAK) 1 and 2, downstream components of the IFN signalling, preserves beta cell function as estimated by mixed-meal–stimulated mean C-peptide level^21^. These recent studies confirm the relevance of TNF-α and IFN-α in the pathogenesis of T1D and their potential as targets to develop future treatments against beta cell malfunction in T1D. Interestingly, *PTPN2* and *BACH2* were also identified as candidate genes for other autoimmune diseases such as Crohn’s disease and rheumatoid arthritis^22–24^, suggesting their important role in the regulation of autoimmunity.

The beta cell outcome following exposure to early-response cytokines is mediated to a great extent by the JAK/STAT and the MAPKs pathways, both triggering different intracellular cascades that may lead to a similar outcome, namely the induction of cell death through the mitochondrial apoptotic pathway^17,25^. IFN-α can activate both pathways while TNF-α is involved in the activation of MAPKs in beta cells^26^, besides activating the NF-κB pathway^27^, but does not induce JAK/STAT. Blocking the MAPKs can inhibit the deleterious effect of IFN-α and TNF-α in beta cells^17^, while blocking the JAK/STAT pathway preserves beta cell viability following exposure to IFNs^25,28^. This suggests that the MAPKs pathway can modulate the intrinsic IFN signaling cascade through crosstalk with JAK/STAT, via mechanisms that remain to be clarified.

Departing from these previous observations, we presently evaluated the implications of the T1D candidate genes *PTPN2* and *BACH2* on the regulation of the JAK/STAT and MAPKs pathways in human islet cells and how this directly modulates ISRE-mediated transcription. We clarified the dynamics of STAT1 activation in human beta cells and proposed a new model for its regulation involving a genetically-regulated crosstalk between the JAK/STAT and the MAPKs pathways.

## Results

### BACH2 modulates cytokine-induced PTPN2 expression in human beta cells.

**Figure 1:**
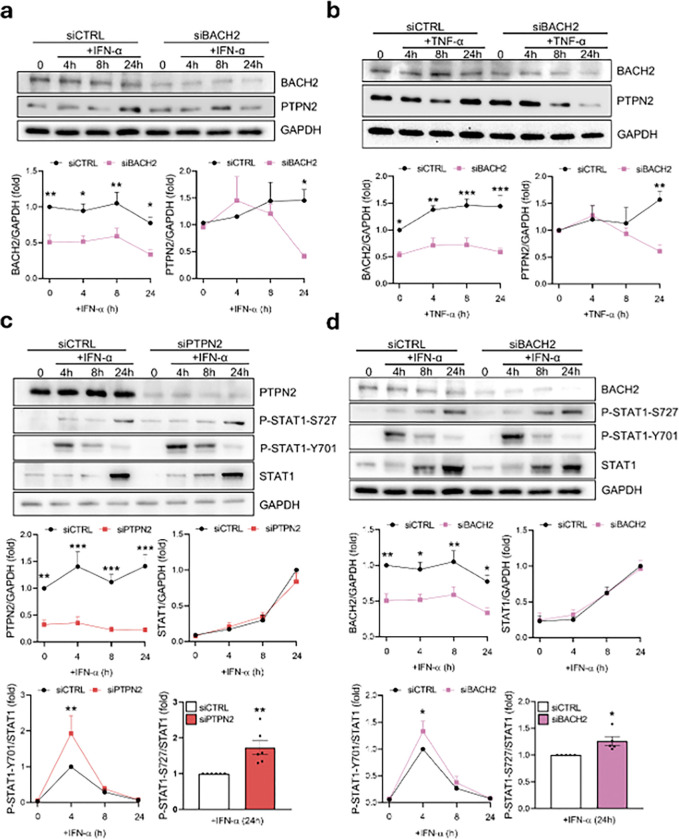
BACH2 and PTPN2 regulate STAT1 activity. (a) and (b) EndoC-βH1 cells were transfected with siCTRL or siBACH2. After 48 h of recovery the cells were left untreated (0) or treated for 4h, 8h, or 24h with IFN-α (2,000 units/ml) (a), or TNF-α (1,000 units/ml) (b). BACH2, PTPN2, and GAPDH protein levels were analyzed by immunoblot. Results are means ± SEM of four to five independent experiments. BACH2 and PTPN2 bands were quantified by densitometry and corrected by GAPDH and presented as fold-variation compared to untreated cells, considered as 1. *p < 0.05, and **p < 0.01 siCTRL vs. siBACH2. ANOVA. (c) EndoC-βH1 cells were transfected with siCTRL or siPTPN2. After 48 h of recovery the cells were left untreated (0) or treated for 4h, 8h, or 24h with IFN-α (2,000 units/ml). PTPN2, P-STAT1-S727, P-STAT1-Y701, STAT1 and GAPDH protein levels were analyzed by immunoblot. Results are means ± SEM of six independent experiments. Bands were quantified by densitometry and corrected by GAPDH for PTPN2 and STAT1; and corrected by STAT1 for P-STAT1-S727 and P-STAT1-Y701. Results are presented as fold-variation compared to untreated cells, considered as 1. **p < 0.01 siCTRL vs. siPTPN2. ANOVA. (d) EndoC-βH1 cells were transfected with siCTRL or siBACH2. After 48 h of recovery the cells were left untreated (0) or treated for 4h, 8h, or 24h with IFN-α (2,000 units/ml). BACH2, P-STAT1-S727, P-STAT1-Y701, STAT1 and GAPDH protein levels were analyzed by immunoblot. Results are means ± SEM of four independent experiments. Bands were quantified by densitometry and corrected by GAPDH for BACH2 and STAT1; and corrected by STAT1 for P-STAT1-S727 and P-STAT1-Y701. Results are presented as fold-variation compared to untreated cells, considered as 1. **p < 0.01 siCTRL vs. siBACH2. ANOVA.

We previously reported that BACH2 silencing modulates PTPN2 expression in human beta cells exposed to IL-1β + IFN-γ^14^. To confirm this effect following a single cytokine (i.e. IFN-α or TNF-α) treatment, we silenced BACH2 in EndoC-βH1 cells (Fig. 1) or iPSC-derived islet-like cells (Supplementary Fig. 1) and treated the cells with IFN-α or TNF-α at different time points. BACH2 silencing significantly reduced PTPN2 expression after 24h treatment in both cell models exposed to IFN-α (72% reduction, p < 0.05) (Fig. 1a and Supplementary Fig. 1b) or TNF-α (71% reduction in EndoC-βH1 cells, p < 0.01; and 53% reduction in iPSC-derived islet-like cells) (Fig. 1b and Supplementary Fig. 1b).

### BACH2 and PTPN2 regulate STAT1 total activity.

PTPN2 deficiency in human beta cells has been reported to affect STAT1 canonical activation through direct tyrosine de-phosphorylation (Y701)^8,17,29^. Thus, BACH2 deficiency may also affect STAT1 activation through PTPN2 modulation^14^. In line with this hypothesis, we observed a significant increase in P-STAT1-Y701 at 4h after IFN-α treatment in EndoC-βH1 cells silenced for PTPN2 (93% increase, p < 0.01) (Fig. 1c) or BACH2 (27% increase, p < 0.05) (Fig. 1d); however, P-STAT1-Y701 decreased rapidly and there were no significant differences against the control condition after 24h of treatment. In iPSC-derived islet-like cells silenced for PTPN2 there was a substantial increase of P-STAT1-Y701 after 24 h treatment with IFN-α (56%, p < 0.01) (Supplementary Fig. 1a), but this effect was not detected in cells silenced for BACH2 (Supplementary Fig. 1b).

STAT1 activation requires not only phosphorylation at Y701 but also phosphorylation at its serine residue (S727), which stabilises DNA binding and enhances the transcriptional activity of STAT1 by actively recruiting additional transcriptional coactivators to the promoters of STAT1 target genes^30,31^. To understand IFN-α-stimulated STAT1 activation in human beta cells, we exposed EndoC-βH1 cells to IFN-α at different time points. STAT1 was first phosphorylated at its Y701 residue with a maximum activation peak at 4h followed by a progressive decrease up to 72h of treatment (Supplementary Fig. 2a, b). On the other hand, STAT1-S727 phosphorylation increased progressively over time having not yet reached its peak even after 72h of IFN-α treatment (Supplementary Fig. 2a, b). Interestingly, this effect appears to be specific for IFN-α, as it was not reproduced in cells exposed to IFN-γ (Supplementary Fig. 2c, d), where both STAT1-Y701 and STAT1-S727 phosphorylation remain up-regulated after 24h of treatment. As STAT1-S727 has been reported to enhance transcriptional activation, we tested the capacity of human beta cells to keep STAT1 activation even after removing IFN-α from the medium; STAT1-Y701 phosphorylation was immediately abolished 8h after the treatment was stopped, while STAT1-S727 phosphorylation was detectable even after 48h (Supplementary Fig. 2e). These results suggest that STAT1-S727 contributes to preserving long-term STAT1 activation in human beta cells.

We next tested STAT1 phosphorylation status in our models of PTPN2 or BACH2 deficiency, observing that EndoC-βH1 cells silenced for PTPN2 (Fig. 1c) or BACH2 (Fig. 1d) had a significant increase in STAT1-S727 after 24h of treatment with IFN-α (67% for PTPN2-silenced cells, p < 0.01; and 25% in BACH2-silenced cells, p < 0.05); a similar outcome was observed in iPSC-derived islet-like cells silenced for PTPN2 (48%, p < 0.01) (Supplementary Fig. 1a) and for BACH2 (53%, p < 0.01) (Supplementary Fig. 1b).

**Figure 2:**
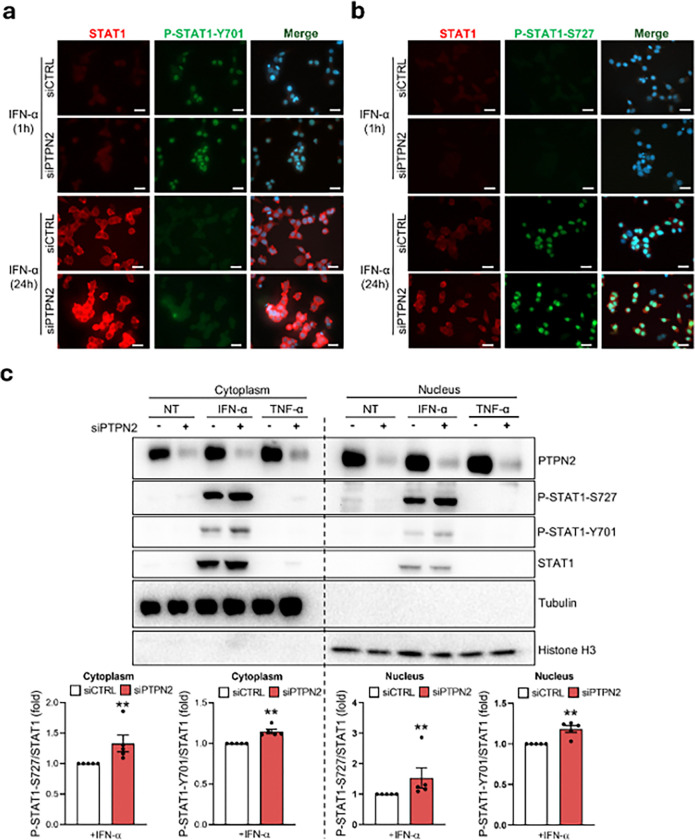
STAT1 location in the nucleus of human beta cells is regulated by PTPN2 and is residue- and time-dependent. (a) and (b) EndoC-βH1 cells were transfected with siCTRL or siPTPN2. After 48 h of recovery the cells were left untreated (0) or treated for 1h or 24h with IFN-α (2,000 units/ml). P-STAT1-S727, P-STAT1-Y701, and STAT1 protein expression were analyzed by wide-field fluorescence microscopy. Images are representative of three independent experiments. (c) EndoC-βH1 cells were transfected with siCTRL or siPTPN2. After 48 h of recovery the cells were left untreated (0) or treated for 24h with IFN-α (2,000 units/ml). Cytoplasmic and nuclear protein fractions were separated and PTPN2, P-STAT1-S727, P-STAT1-Y701, STAT1, tubulin and histone H3 protein levels were analyzed by immunoblot. Results are means ± SEM of five independent experiments. P-STAT1-S727 and P-STAT1-Y701 bands were quantified by densitometry and corrected by STAT1. Results are presented as fold-variation compared to untreated cells, considered as 1. **p < 0.01 siCTRL vs. siPTPN2. ANOVA. Tubulin was used as a positive control for the cytoplasmic fraction. Histone H3 was used as a positive control for the nuclear fraction.

To better understand the impact of the different STAT1 phosphorylation in human beta cells, we focused on IFN-α-induced STAT1 nuclear translocation. STAT1-Y701 phosphorylation was mainly located in the nucleus after 1h of IFN-α exposure (Fig. 2a) but this phosphorylated form was barely detected after 24h of treatment and the remaining STAT1-Y701 was mainly observed in the cytoplasm (Fig. 2c). In contrast, STAT1-S727 phosphorylation was only detected in the nucleus after 24h (Fig. 2b, c). Silencing of PTPN2 increased STAT1-Y701 and S727 phosphorylation both in the nucleus (18% for STAT1-Y701, p < 0.01; and 42% for STAT1-S727, p < 0.01) and in the cytoplasm (15% for STAT1-Y701, p < 0.01; and 31% for STAT1-S727, p < 0.01) (Fig. 2c).

**Figure 3:**
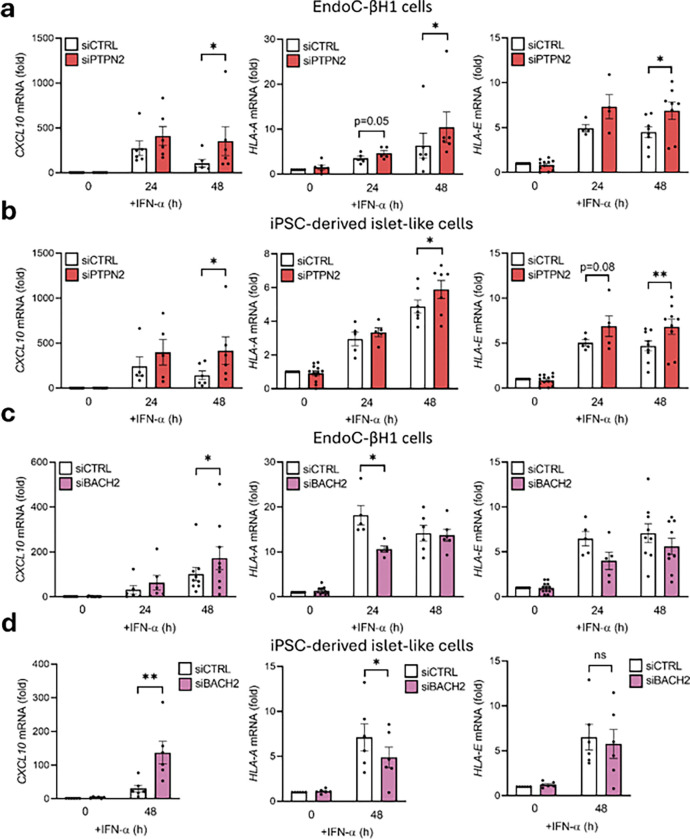
PTPN2 and BACH2 regulate IFN-α-stimulated gene expression in human beta cells. EndoC-βH1 (a) and iPSC-derived islet-like cells (b) were transfected with siCTRL (white bars) or siPTPN2 (red bars). After 48 h of recovery cells were left untreated (0) or treated for 24h, or 48h with IFN-α (2,000 units/mL). *CXCL10*, *HLA-A*, and *HLA-E*levels were analyzed by qRT-PCR. mRNA expression was normalized to the geometrical mean of *ACTB* and *VAPA* and presented as fold-variation compared with siCTRL-untreated cells, considered as 1. Results are means ± SEM of four to eight independent experiments. *p < 0.05, and **p < 0.01 vs siCTRL. EndoC-βH1 (c) and iPSC-derived islet-like cells (d) were transfected with siCTRL (white bars) or siBACH2 (pink bars). After 48 h of recovery cells were left untreated (0) or treated for 24h, or 48h with IFN-α (2,000 units/mL). *CXCL10*, *HLA-A*, and *HLA-E* levels were analyzed by qRT-PCR. mRNA expression was normalized to the geometrical mean of *ACTB* and *VAPA*and presented as fold-variation compared with siCTRL-untreated cells, considered as 1. Results are means ± SEM of four to eight independent experiments. *p < 0.05 vs siCTRL.

We hypothesized that the longer retention of STAT1 in the nucleus through its S727 residue could be translated into an increased gene expression after long-time exposure to IFN-α. To test this, we silenced EndoC-βH1 cells for PTPN2 or BACH2 and assayed the transcription of selected ISRE-mediated genes (*CXCL10*, *HLA-A*, and *HLA-E*) after 24h or 48h of IFN-α exposure. *CXCL10* expression was significantly increased in PTPN2- or BACH2-silenced cells after 48h (158% for PTPN2, p < 0.05; 41% for BACH2, p < 0.05) (Fig. 3a, c) and its expression was higher or maintained in comparison with cells treated for 24h only. In an analogous experiment performed in iPSC-derived islet-like cells, we observed a similar outcome for *CXCL10* expression (192% for PTPN2, p < 0.05; 411% for BACH2, p < 0.01) (Fig. 3b, d). *HLA-A* and *HLA-E* expression showed a similar profile as *CXCL10* expression in EndoC-βH1 cells silenced for PTPN2 (116% increase for *HLA-A*, p < 0.01; 50% increase for *HLA-E*, p < 0.01) (Fig. 3a) and iPSC-derived islet-like cells (20% increase for *HLA-A*, p < 0.05; 44% increase for *HLA-E*, p < 0.01) (Fig. 3b). BACH2 silencing, however, led to an opposite profile with reduced expression of *HLA-A* in EndoC-βH1 cells after 24h exposure (41% decrease, p < 0.05) (Fig. 3c) and iPSC-derived islet-like cells after 48h exposure (37% decrease, p < 0.05) (Fig. 3d), while no significant changes were detected on *HLA-E* expression after 48h exposure to IFN-α.

BACH2 is a transcription factor, but there are no binding sites for BACH2 in the *PTPN2* gene ^14^. Given that a majority of BACH2 binding sites are outside annotated promoter regions^32^, we explored the possibility of BACH2 acting through an intermediate gene by analysing RNA-Seq datasets on different pancreatic tissues to predict novel binding sites for the BACH2 motif (Supplementary Fig. 3a). We identified 56 candidate genes common to all databases with a potential association with BACH2 (Supplementary Fig. 3b,c). From those, several genes (e.g. *STK40*, *CD81*, or *TLR4*) have been associated with NF-kB regulation^33–35^. According to this, the functional enrichment for the target genes of BACH2 in the hTFtarget database showed a significant modulation of pathways associated with NF-kB (Supplementary Table 4). These observations suggest that BACH2 impacts beta cells not only via PTPN2 regulation, but also via other pathways potentially related to NF-kB.

### STAT1-Y701 and STAT1-S727 are essential to initiate the IFN-α transcriptional program.

To better understand the role of the two STAT1 phosphorylation residues we switched to a HeLa cell model devoid of STAT1, enabling transfection of STAT1 molecules with mutations either in the S727 residue (Stat1 alpha S727A pRc/CMV)^36^ or in the Y701 residue (pLV-Y701F-STAT1) ^37^. We first validated the ability of wild-type (wt) HeLa cells to respond to IFN-α, similar to the above-described findings in human beta cells (Fig. 1, 3). As observed for human beta cells, Hela cells exposed to IFN-α increased P-STAT2 and STAT1 total protein expression (Supplementary Fig. 4a). They were also capable of increasing *HLA-A*, *CXCL10* and *PDL1* mRNA expression following exposure to IFN-α (Supplementary Fig. 4b) (we have previously shown that IFN-α up-regulates PDL1 expression in human beta cells^38^). HeLa cells also express *PTPN2* and its silencing induced an increase in P-STAT1-Y701 following exposure to IFN-α (41% increase, p < 0.05) (Supplementary Fig. 4c).

**Figure 4:**
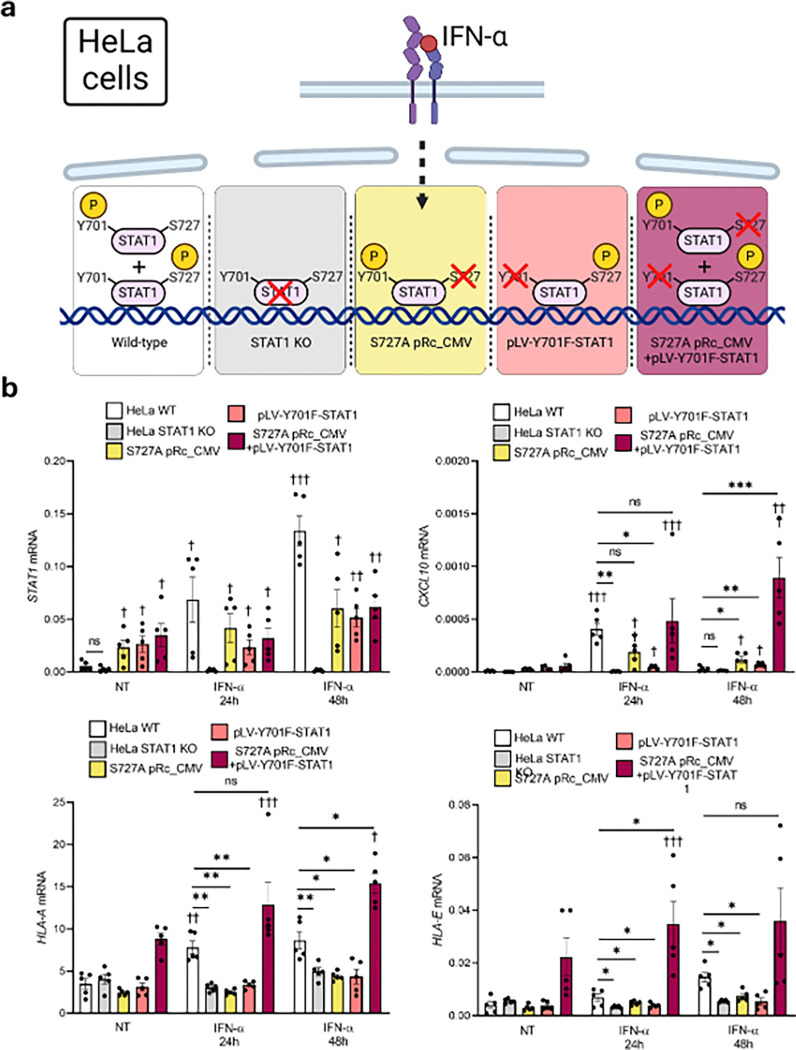
The activation of the different STAT1 phosphorylation residues show independent effects on IFN-α-induced gene expression. (a) HeLa cells knock-out for STAT1 were transfected with the pLV-Y701F-STAT1 plasmid, the S1α S727A plasmid, or the mixture 1:1 of both plasmids. After 48 h of recovery cells were left untreated (0) or treated for 1h, or 24h with IFN-α (2,000 units/mL). (b) *STAT1*, *CXCL10*, *HLA-A*, and *HLA-E* levels were analyzed by qRT-PCR. mRNA expression was normalized to the geometrical mean of *ACTB*. Results are means ± SEM of five independent experiments. *p < 0.05, **p < 0.01, vs wild-type-treated HeLa cells; ^†^p < 0.05, ^††^p < 0.01, ^†††^p < 0.001, vs knock-out-treated HeLa cells; ANOVA. Wild-type HeLa cells were used as a positive control. Non-transfected STAT1 knock-out HeLa cells were used as a negative control. Panel (a) created with BioRender.com

To characterize the specific effects of the different STAT1 phosphorylation residues, we transfected HeLa cells knocked out for STAT1 with plasmids containing STAT1 with a mutation on the S727 residue (Stat1 alpha S727A pRc/CMV)^36^, a mutation on the Y701 residue (pLV-Y701F-STAT1)^37^ or a mixture of both plasmids (Fig. 4a, supplementary Fig. 5a, b). We then exposed the cells expressing the mutated STAT1 forms for 24h or 48h with IFN-α and measured the gene expression of selected IFN-α-stimulated genes (*CXCL10*, *HLA-A*, and *HLA-E*). Interestingly, *CXCL10* expression was independently induced by both STAT1 residues, while *HLA-A* and *HLA-E* were not induced by phosphorylation of each residue alone (Fig. 4b). However, *CXCL10* and *HLAs* expression was restored when both mutated plasmids were present.

*CXCL10* expression is partially inhibited in normal conditions after longer exposures to IFN-α for HeLa cells (94% inhibition 48h vs 24h IFN-α treatment, p < 0.05) (Fig. 4b); this phenomenon was also observed in beta cells (Fig. 3a, b; Fig. 9c). However, when we transfected both mutated plasmids in HeLa cells, in a context where cells present STAT1 molecules with the capacity of being phosphorylated in only one of their active residues, this partial inhibition was abolished and cells continued to increase *CXCL10* expression after 24h. These results suggest that the phosphorylation of different residues in a single STAT1 molecule could be exclusive and self-inhibitory.

### STAT1-Y701 and STAT1-S727 phosphorylation can be dissociated, showing a novel crosstalk in human beta cells between the JAK/STAT and the MAPKs pathway.

We next questioned whether the regulation of STAT1-Y701 and STAT1-S727 have independent impacts on the control of gene expression in human beta cells. We focused on the two main intracellular pathways activated by IFN-α, the JAK/STAT and the MAPKs pathway. The first one is responsible for the canonical STAT1 signalling pathway that leads to the activation of the ISRE-mediated gene expression^25^, while the MAPKs pathway is mostly involved in STAT1-independent pathogenic effects of IFN-α^17^. There are, however, reports suggesting the participation of members of the MAPKs pathway in STAT1 activation in other cell types^39,40^.

**Figure 5:**
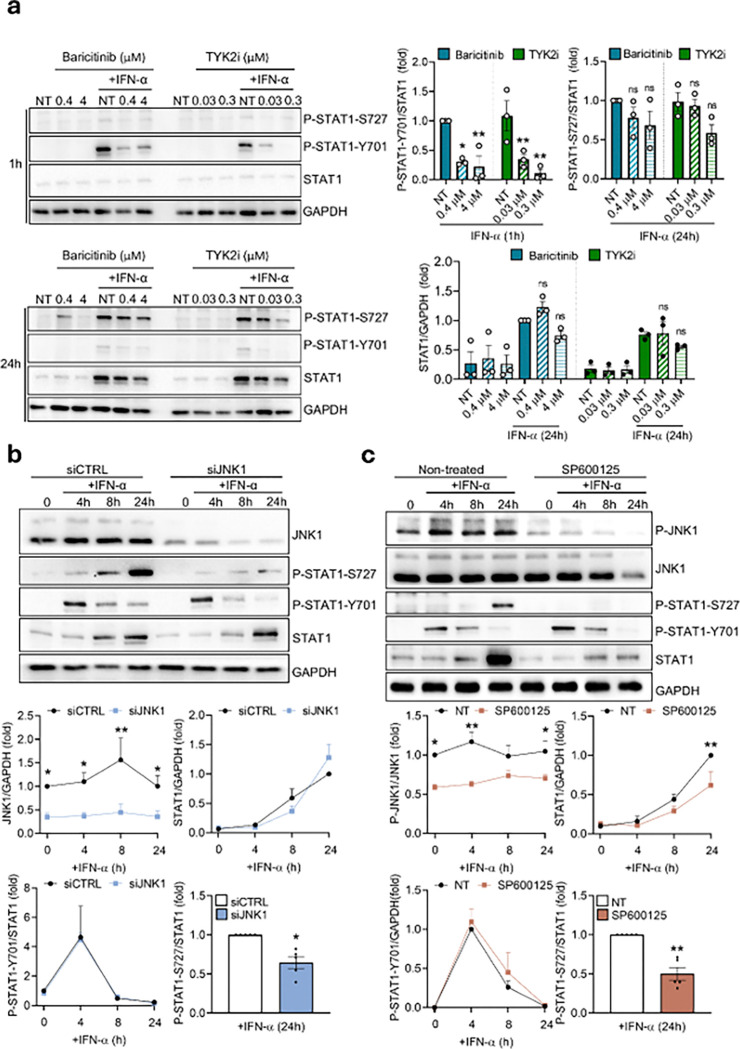
The activation of the different STAT1 residues is independent, pathway-specific and can be dissociated in human beta cells. (a) EndoC-βH1 cells were pre-treated for 2h with 0, 0.4 or 4 μM Baricitinib; or 0, 0.03 or 0.3 μM TYK2 inhibitor (BMS-986165). Then, cells were left untreated (0) or treated for 1h or 24h with IFN-α (2,000 units/ml) with or without the corresponding inhibitor. P-STAT1-S727, P-STAT1-Y701, STAT1 and GAPDH protein levels were analyzed by immunoblot. Results are means ± SEM of three independent experiments. Bands were quantified by densitometry and corrected by GAPDH for STAT1 or corrected by STAT1 for P-STAT1-S727 and P-STAT1-Y701. Results are presented as fold-variation compared to control IFN-α-exposed cells, considered as 1. *p < 0.05, and **p < 0.01 vs. control. ANOVA. ns: non-significant. (b) EndoC-βH1 cells were transfected with siCTRL or siJNK1. After 48 h of recovery the cells were left untreated (0) or treated for 4h, 8h, or 24h with IFN-α (2,000 units/ml). JNK1, P-STAT1-S727, P-STAT1-Y701, STAT1 and GAPDH protein levels were analyzed by immunoblot. Results are means ± SEM of five independent experiments. Bands were quantified by densitometry and corrected by GAPDH for JNK1 and STAT1 or corrected by STAT1 for P-STAT1-S727 and P-STAT1-Y701. Results are presented as fold-variation compared to untreated cells, considered as 1. *p < 0.05, **p < 0.01 siCTRL vs. siJNK1. ANOVA. (c) EndoC-βH1 cells were pre-treated for 2h with 20 μM JNK inhibitor SP600125. Then, cells were left untreated (0) or treated for 4, 8, or 24h with IFN-α (2,000 units/ml) with or without SP600125. P-JNK1, JNK1, P-STAT1-S727, P-STAT1-Y701, STAT1 and GAPDH protein levels were analyzed by immunoblot. Results are means ± SEM of five independent experiments. Bands were quantified by densitometry and corrected by GAPDH for STAT1; corrected by STAT1 for P-STAT1-S727 and P-STAT1-Y701; or corrected by JNK for P-JNK1. Results are presented as fold-variation compared to untreated cells, considered as 1. **p < 0.01 vs. control; ANOVA.

We first blocked the two main downstream kinases in the JAK/STAT pathway by using a JAK1/2 inhibitor (Baricitinib) or a TYK2 inhibitor (BMS-986165). EndoC-βH1 cells treated with the inhibitors and exposed to 1h or 24h of IFN-α showed a dose-dependent reduction of STAT1-Y701 phosphorylation, reducing its activation by 70% with 0.4 μM Baricitinib (p < 0.05) and by 90% with 4 μM Baricitinib (p < 0.01). There was a reduction of STAT1-Y701 phosphorylation by 68% with 0.03 μM TYK2 inhibitor (p < 0.01) and by 92% with 0.3 μM TYK2 inhibitor (p < 0.01). However, both inhibitors failed to suppress STAT1-S727 phosphorylation or to completely abolish IFN-α-induced STAT1 total expression (Fig. 5a).

As the JAK/STAT pathway showed little effect on IFN-α-mediated STAT1-S727 phosphorylation, we questioned if specific members of the MAPKs pathway could be the main responsible for this process. We first focused on JNK1, as we have observed in a previous study its involvement in the pathogenic effects of IFN-α and its modulation by PTPN2 via de-phosphorylation^17^. When we silenced EndoC-βH1 cells for JNK1 and exposed them to IFN-α, STAT1-S727 phosphorylation was reduced after 24h of exposure (37%, p < 0.05), while no differences in STAT1-Y701 phosphorylation or IFN-α-induced STAT1 total expression were detected at any time points assayed (Fig. 5b); we observed a similar result in iPSC-derived islet-like cells (49%, p < 0.05) (Supplementary Fig. 6). To confirm the involvement of the JNKs on STAT1 activation, we tested a chemical inhibitor for all three JNKs (SP600125); EndoC-βH1 cells exposed to IFN-α in the presence of the inhibitor decrease STAT1-S727 phosphorylation (58%, p < 001) but also IFN-α-induced STAT1 total expression (49%, p < 0.01), without affecting STAT1-Y701 phosphorylation (Fig. 5c).

**Figure 6:**
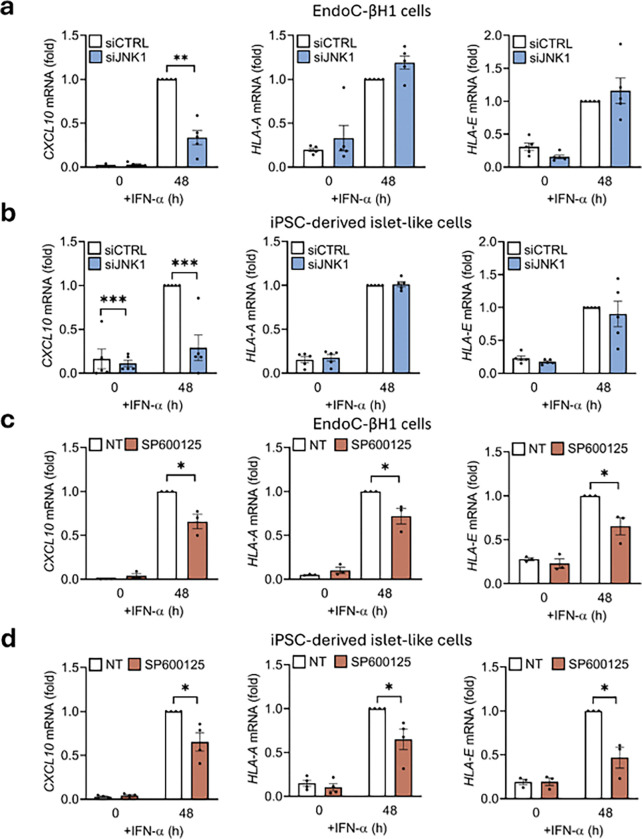
The MAPK pathway regulates IFN-α-stimulated gene expression in human beta cells. EndoC-βH1 (a) and iPSC-derived islet-like cells (b) were transfected with siCTRL (white bars) or siJNK1 (blue bars). After 48 h of recovery cells were left untreated (0) or treated for 48h with IFN-α (2,000 units/mL). *CXCL10*, *HLA-A*, and *HLA-E*levels were analyzed by qRT-PCR. mRNA expression was normalized to the geometrical mean of *ACTB* and *VAPA* and presented as fold-variation compared with siCTRL-IFN-α-treated cells, considered as 1. Results are means ± SEM of five independent experiments. *p < 0.05, and ***p < 0.001 vs siCTRL. EndoC-βH1 (c) and iPSC-derived islet-like cells (d) were pre-treated for 2h with 20 μM JNK inhibitor SP600125. Then, cells were left untreated (0) or treated for 48h with IFN-α (2,000 units/ml) with (brown bars) or without SP600125 (white bars). *CXCL10*, *HLA-A*, and *HLA-E*levels were analyzed by qRT-PCR. mRNA expression was normalized to the geometrical mean of *ACTB* and *VAPA* and presented as fold-variation compared with siCTRL IFN-α-treated cells, considered as 1. Results are means ± SEM of three to four independent experiments. *p < 0.05 vs siCTRL.

As blocking the MAPKs showed no significant changes in the canonical STAT1 activation through Y701, we then questioned if gene expression could still be affected. Thus, we silenced or chemically blocked JNK1 activation and measured the gene expression of selected IFN-α-stimulated genes (*CXCL10*, *HLA-A*, and *HLA-E*) after 48h exposure to IFN-α. EndoC-βH1 (Fig. 6a) and iPSC-derived islet-like cells (Fig. 6b) silenced for JNK1 showed a significant decrease in *CXCL10* expression (71% in EndoC-βH1 cells, p < 0.05; and a 90% in iPSC-derived islet-like cells, p < 0.001); but no differences on *HLA-A* or *HLA-E* expression were detected. However, when EndoC-βH1 cells (Fig. 6c) or iPSC-derived islet-like cells (Fig. 6d) were exposed to IFN-α in the presence of the chemical inhibitor SP600125 there was a significant decrease in *CXCL10* (35% in EndoC-βH1 cells, p < 0.05; and a 47% in iPSC-derived islet-like cells, p < 0.05), *HLA-A* (29% in EndoC-βH1 cells, p < 0.05; and a 39% in iPSC-derived islet-like cells, p < 0.05), and *HLA-E* expression (36% in EndoC-βH1 cells, p < 0.05; and a 57% in iPSC-derived islet-like cells, p < 0.05); supporting the significant decrease observed in total STAT1 in cells treated with the SP600125 inhibitor (Fig. 5c), that is not reproduced when cells are only silenced for JNK1 (Fig. 5b).

### p38 MAPK is a key player in STAT1 activation and its phosphorylation is regulated by PTPN2 and BACH2.

**Figure 7:**
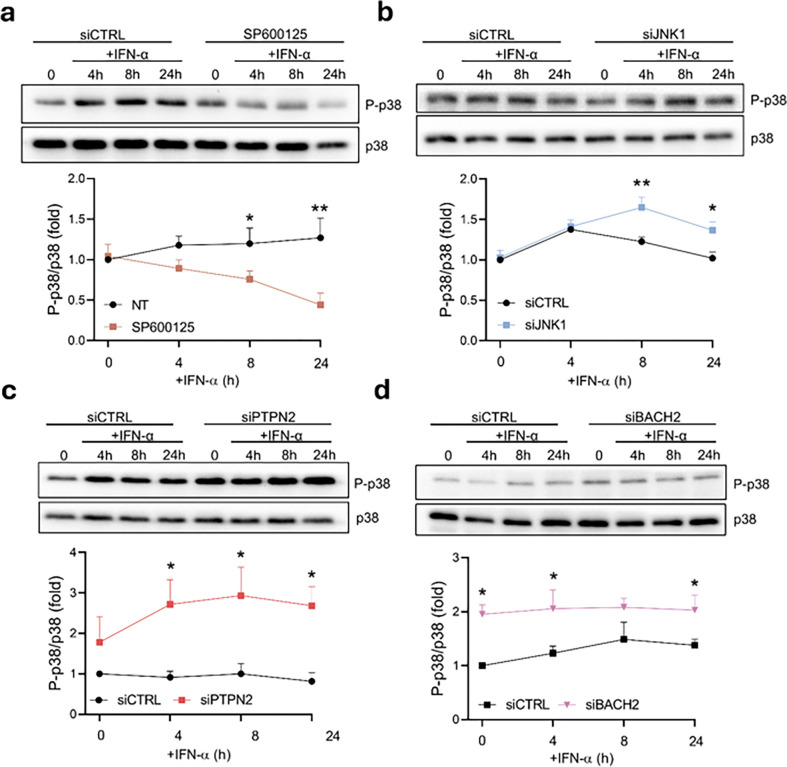
PTPN2 or BACH2 silencing induces an overactivation of p38 MAPK in human beta cells. (a) EndoC-βH1 cells were pre-treated for 2h with 20 μM JNK inhibitor SP600125. Then, cells were left untreated (0) or treated for 4, 8, or 24h with IFN-α (2,000 units/ml) with or without SP600125. P-p38 and p38 protein levels were analyzed by immunoblot. Results are means ± SEM of three independent experiments. Bands were quantified by densitometry and corrected by p38. Results are presented as fold-variation compared to untreated cells, considered as 1. *p < 0.05, **p < 0.01 vs. control; ANOVA. (b) EndoC-βH1 cells were transfected with siCTRL or siJNK1. After 48 h of recovery the cells were left untreated (0) or treated for 4h, 8h, or 24h with IFN-α (2,000 units/ml). P-p38 and p38 protein levels were analyzed by immunoblot. Results are means ± SEM of four independent experiments. Bands were quantified by densitometry and corrected by p38. Results are presented as fold-variation compared to untreated cells, considered as 1. *p < 0.05, **p < 0.01 siCTRL vs. siJNK1; ANOVA. (c) EndoC-βH1 cells were transfected with siCTRL or siPTPN2. After 48 h of recovery the cells were left untreated (0) or treated for 4h, 8h, or 24h with IFN-α (2,000 units/ml). P-p38 and p38 protein levels were analyzed by immunoblot. Results are means ± SEM of four independent experiments. Bands were quantified by densitometry and corrected by p38. Results are presented as fold-variation compared to untreated cells, considered as 1. *p < 0.05 siCTRL vs. siPTPN2; ANOVA. (d) EndoC-βH1 cells were transfected with siCTRL or siBACH2. After 48 h of recovery the cells were left untreated (0) or treated for 4h, 8h, or 24h with IFN-α (2,000 units/ml). P-p38 and p38 protein levels were analyzed by immunoblot. Results are means ± SEM of four independent experiments. Bands were quantified by densitometry and corrected by p38. Results are presented as fold-variation compared to untreated cells, considered as 1. *p < 0.05 siCTRL vs. siBACH2; ANOVA.

The chemical inhibition of the JNKs was more effective on STAT1 regulation than JNK1 silencing itself, which made us question whether the other JNK forms were also affecting STAT1 activation dynamics or whether the inhibitor induced non-target effects on another member of the MAPKs pathway. As p38 MAPK was associated in previous studies with STAT1-S727 phosphorylation^39^, we assayed p38 MAPK activation levels on EndoC-βH1 cells exposed to IFN-α; to our surprise, p38 MAPK phosphorylation was inhibited in the presence of the JNKs inhibitor SP600125 by a 68% after 24h treatment (p < 0.01) (Fig. 7a), but not in cells silenced for JNK1, where we even observed a 34% increase after 24 h treatment with IFN-α (p < 0.05) (Fig. 7b).

p38 MAPK has been suggested as a substrate for PTPN2 in other cell types^41^. We thus analyzed the effect of the absence of PTPN2 or BACH2 in EndoC-βH1 cells on p38 MAPK phosphorylation, showing a significant increase of p38 MAPK activation in cells exposed to IFN-α and silenced for PTPN2 (259%, p < 0.05) (Fig. 7c) or BACH2 (44%, p < 0.01) (Fig. 7d) after 24h treatment; interestingly, BACH2 silencing appears to induce a constitutive increase in P-p38 in non-treated cells (94%, p < 0.05). A similar effect was observed in iPSC-derived islet-like cells, where PTPN2 silencing increased P-p38 after 24h treatment with IFN-α (45%, p < 0.05) (Supplementary Fig. 7a), while BACH2 silencing produced an IFN-α-induced increase in P-p38 (44%, p < 0.05) and showed a clear tendency to constitutively induce p38 MAPK activation (p < 0.08) (Supplementary Fig. 7b).

**Figure 8:**
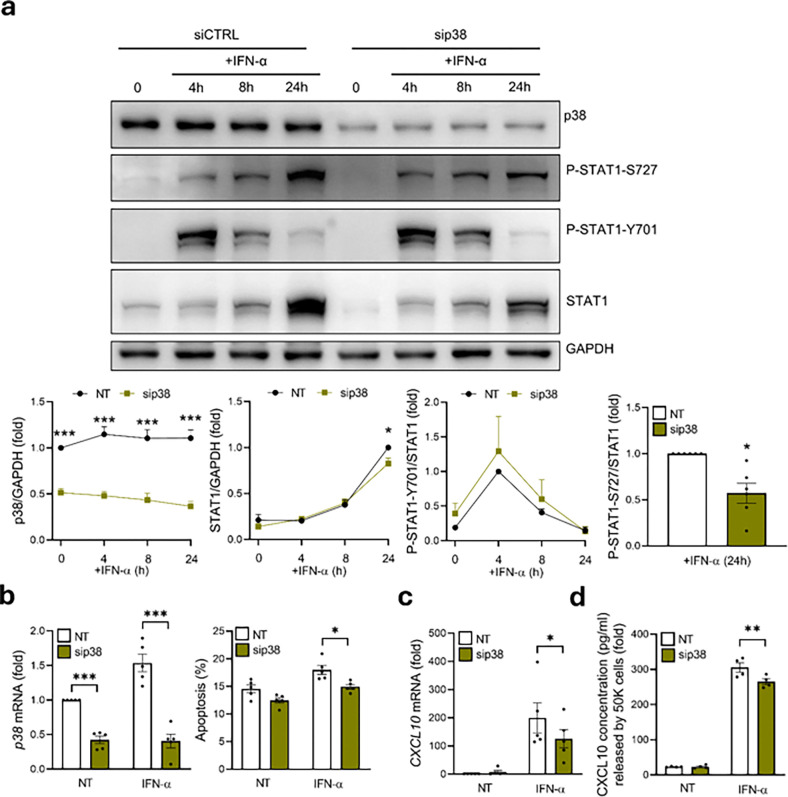
p38 MAPK regulates STAT1-S727 activation, beta cell resistance to IFN-α-mediated apoptosis and CXCL10 production. (a) EndoC-βH1 cells were transfected with siCTRL or sip38. After 48 h of recovery the cells were left untreated (0) or treated for 4h, 8h, or 24h with IFN-α (2,000 units/ml). p38, P-STAT1-S727, P-STAT1-Y701, STAT1 and GAPDH protein levels were analyzed by immunoblot. Results are means ± SEM of six independent experiments. Bands were quantified by densitometry and corrected by GAPDH for p38 and STAT1 or corrected by STAT1 for P-STAT1-S727 and P-STAT1-Y701. Results are presented as fold-variation compared to untreated cells, considered as 1. *p < 0.05, ***p < 0.001 siCTRL vs. sip38. ANOVA. (b) EndoC-βH1 cells were transfected with siCTRL (white bars) or sip38 (gold bars). After 48 h of recovery, cells were left untreated (NT) or treated for 48 h with IFN-α (2000 U/ml). siP38 silencing was confirmed by qRT-PCR. mRNA expression was normalised to the geometric mean of *ACTB* and *VAPA*and presented as fold-variation compared with siCTRL-untreated cells, considered as 1. Results are means ± SEM of five independent experiments. *p < 0.05 vs siCTRL. Cell death was evaluated using Hoechst and propidium iodide staining. Results are means ± SEM of six to eight independent experiments. *p<0.05 and vs siCTRL; ANOVA. (c) EndoC-βH1 cells were transfected with siCTRL or sip38. After 48 h of recovery the cells were left untreated (0) or treated for 24h with IFN-α (2,000 units/ml). *CXCL10* levels were analyzed by qRT-PCR. mRNA expression was normalized to the geometrical mean of *ACTB*and *VAPA* and presented as fold-variation compared with siCTRL-untreated cells, considered as 1. Results are means ± SEM of four independent experiments. *p < 0.05 vs siCTRL; ANOVA (d) EndoC-βH1 cells were transfected with siCTRL or sip38. After 48 h of recovery the cells were left untreated (0) or treated for 24h with IFN-α (2,000 units/ml). CXCL10 secretion levels were analyzed by ELISA. Results are presented as fold-variation compared with siCTRL-untreated cells, considered as 1. Results are means ± SEM of four independent experiments. **p < 0.01 vs siCTRL; ANOVA.

To understand the relevancy of p38 MAPK on human beta cell-IFN-α signalling, we silenced p38 MAPK in EndoC-βH1 cells exposed to IFN-α, observing a similar phenotype to what we detected by using the chemical inhibitor SP600125; with a significant decrease in STAT1-S727 phosphorylation (50%, p < 0.05) and IFN-α-induced STAT1 total expression (18%, p < 0.05), without affecting STAT1-Y701 phosphorylation (Fig. 8a). Finally, p38 MAPK silencing was also able to partially protect EndoC-βH1 cells to IFN-α-mediated apoptosis (17%, p < 0.05) (Fig. 8b), and inhibited *CXCL10* expression (39%, p < 0.05) (Fig. 8c) and secretion (13%, p < 0.01) (Fig. 8d).

### The regulation of the JAK/STAT and MAPKs pathways is also essential to control IFN-α responses in primary human islets.

**Figure 9:**
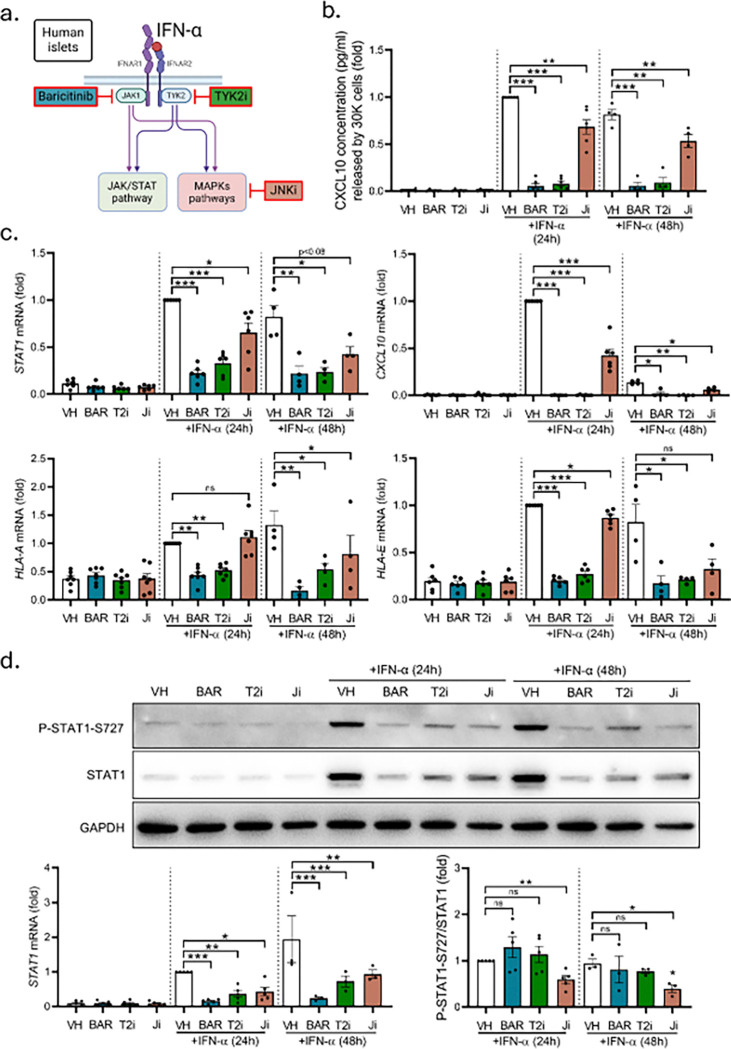
The regulation of the JAK/STAT and MAPKs pathways is essential to control IFN-α responses in primary human islets. (a) Dispersed primary human islets were pre-treated for 2h with 4 μM Baricitinib; or 0.3 μM TYK2 inhibitor (BMS-986165); or 20 μmol/l JNK inhibitor (SP600125). Then, cells were left untreated (0) or treated for 24h or 48h with IFN-α (2,000 units/ml) with or without the corresponding inhibitor. (b) CXCL10 secretion levels were analyzed by ELISA. Results are presented as fold-variation compared with vehicle 24h IFN-α-treated cells, considered as 1. **p < 0.01, ***p < 0.001 vs IFN-α vehicle-treated control. ANOVA. (c) *STAT1*, *CXCL10*, *HLA-A*, and *HLA-E*mRNA levels were analyzed by qRT-PCR. mRNA expression was normalized to the geometrical mean of *ACTB* and *VAPA* and presented as fold-variation compared with vehicle 24h IFN-α-treated cells, considered as 1. *p < 0.05, **p < 0.01, and ***p < 0.001 vs IFN-α vehicle-treated control; ANOVA. (d) P-STAT1-S727, STAT1 and GAPDH protein levels were analyzed by immunoblot. Bands were quantified by densitometry and corrected by GAPDH for STAT1 or corrected by STAT1 for P-STAT1-S727. Results are presented as fold-variation compared with vehicle 24h IFN-α-treated cells, considered as 1. *p < 0.05, **p < 0.01, and ***p < 0.001 vs. IFN-α vehicle-treated control; ANOVA. Results are means ± SEM obtained from three to six healthy donors. (BAR: Baricitinib; T2i: TYK2 inhibitor; Ji: JNK inhibitor; ns: non-significant). Panel (a) created with BioRender.com.

Finally, we treated primary dispersed human islets from healthy donors with Baricitinib or TYK2 inhibitors to block the JAK/STAT pathway, or with the JNK inhibitor to block the MAPKs pathway and then exposed the islet cells to IFN-α for 24h or 48h (Fig. 9a). All three inhibitors were able to decrease IFN-α-induced CXCL10 secretion after 24h IFN-α treatment (97% for Baricitinib, p < 0.001; 94.5% for TYK2 inhibitor, p < 0.001; and a 34% for JNK inhibitor, p < 0.01) and at similar levels after 48h IFN-α treatment (97% for Baricitinib, p < 0.001; 93.5% for TYK2 inhibitor, p < 0.01; and a 36% for JNK inhibitor, p < 0.01) (Fig. 9b). This was in parallel with the decrease observed in *CXCL10* mRNA expression (99.9% for Baricitinib, p < 0.001; 99.8% for TYK2 inhibitor, p < 0.001; and a 60% for JNK inhibitor, p < 0.001, after 24h IFN-α treatment; 98.7% for Baricitinib, p < 0.05; 99.3% for TYK2 inhibitor, p < 0.01; and a 57% for JNK inhibitor, p < 0.05, after 48h IFN-α treatment). For the MHC class I molecules, Baricitinib and TYK2 inhibitors completely inhibited IFN-α-induced *HLA-A* and *HLA-E* mRNA expression. The JNK inhibitor did not affect *HLA-A* expression after 24h IFN-α exposure, but mildly reduced *HLA-E* expression (13%, p < 0.05) (Fig. 9c); however, longer exposures to IFN-α plus the JNK inhibitor reduced *HLA-A* expression by 50% (p < 0.05) in cells treated with the JNK inhibitor. *STAT1* mRNA expression was also inhibited in all three conditions, with a 79% decrease for Baricitinib, p < 0.001; 69% for TYK2 inhibitor, p < 0.001, and a 40% for JNK inhibitor, p < 0.05, after 24h IFN-α treatment; a 77% decrease for Baricitinib, p < 0.01; 72% for TYK2 inhibitor, p < 0.05, and a mild decrease without reaching significance for JNK inhibitor (50%, p < 0.08) in cells treated for 48h with IFN-α (Fig. 9c). These decreases were similar between the three inhibitors at the protein level (86% for Baricitinib, p < 0.001; 69% for TYK2 inhibitor, p < 0.01; and a 62% for JNK inhibitor, p < 0.05 after 24h INF-α treatment; 86% for Baricitinib, p < 0.001; 60% for TYK2 inhibitor, p < 0.001; and a 47% for JNK inhibitor, p < 0.01, after 48h INF-α treatment) (Fig. 9d). Interestingly, even if the three inhibitors were able to decrease the quantity of absolute phosphorylated STAT1-S727 proteins, only the effect of the JNK inhibitors was specific to phosphorylation and not accompanied by an overall reduction of STAT1 (Fig. 9d).

## Discussion

In the present study we show that the T1D candidate genes *PTPN2* and *BACH2* contribute together to the maintenance of the IFN-α signalling in human beta cells through the regulation of STAT1 activation. This is done by acting via two independent pathways, namely the JAK/STAT pathway involved in the short-term activation of STAT1 at its tyrosine residue, and the MAPKs pathway involved in its long-term activation through serine phosphorylation. PTPN2 induces the direct de-phosphorylation of the tyrosine residue of STAT1 and, indirectly through JNK1 and p38 MAPK, the de-phosphorylation of the serine residue of STAT1. On the other hand, BACH2 acts upstream of PTPN2 by regulating its expression. These combined actions provide negative feedback for IFN-α signaling, preventing the overexpression of both proteins involved in immune cell recruitment (e.g. CXCL10) and antigen presentation via the major histocompatibility complex HLA. We also clarified how STAT1 is dynamically regulated by IFN-α and how this dynamic activation impacts downstream gene expression. Indeed, both STAT1 phosphorylated residues, e.g. the tyrosine residue (Y701) and the serine residue (S727), are required for the expression of members of the HLA class I complex, while each residue alone is capable of initiating the expression of *CXCL10*.

STAT1 is a key mediator of IFN-α signalling, and through its interaction with STAT2 and IRF9 it regulates beta cell immune responses^25^, such as the readiness of neighbour cells against viral infections^42^. STAT1 has three major phosphorylation sites, i.e. at its tyrosine (Y701), serine (S727) and threonine residues (T748). T748 is the lesser known, but a recent study has shown that it may function as a general mechanism in macrophages to promote the inflammatory response in an IFN-independent context and even restrict IFN signalling^43^. The canonical STAT1 pathway in response to IFNs mainly involves Y701 as an initiator of the intracellular signal while S727 seems to function as the long-term keeper of the effects of IFNs^36^. In response to IFNs Y701 occurs early in the cytoplasm to assemble the complex with STAT2 and IRF9 and induce its translocation to the nucleus; once there, STAT1 is assembled into chromatin-associated transcriptional complexes and becomes S727-phosphorylated and fully biologically active^44,45^.

Besides the apparent requirement of STAT1 nuclear translocation ahead of S727 phosphorylation, other IFN-independent mechanisms have been shown to activate S727 in response to bacterial lipopolysaccharide, UV irradiation or TNF-α chronic exposure^46^. This phenomenon is mediated by the MAPK member p38 MAPK, but JNK1 was also proposed as an initiator of S727 activation in JB6 Cl 41 mouse epidermal cells^47^. Little is known however about the relevancy of S727 for gene expression in human beta cells and its potential implication in the early beta cell responses contributing to their deleterious dialogue with the immune system in early T1D^2^. Here we show that S727 is directly involved in the transcription of important immune-related genes, such as *CXCL10* or *HLA-A* and demonstrate that two members of the MAPKs pathways, i.e. JNK1 and p38 MAPK, are responsible for S727 phosphorylation. Moreover, we show that Y701 and S727 phosphorylation can be chemically dissociated in human beta cells, opening a window of opportunity for the development of specific treatments targeting one of these pathways to modulate the excessive IFN-α responses in a pro-inflammatory context and thus protect beta cells from the autoimmune attack in T1D^4,48^. In line with our findings, a recent study has associated S727 with the promotion of autoimmune antibody-forming cells and germinal center responses, driving autoantibody production and systemic lupus erythematosus development^49^. Interestingly, we observed that the constitutive decrease in *CXCL10* expression after long-term exposures to IFN-α (48h) in HeLa cells is abolished when both residues are active on independent molecules. This suggests a potentially novel self-regulatory pattern for STAT1, present when an individual STAT1 molecule is not phosphorylated in both residues at the same time. This possibility requires additional studies.

The risk of developing T1D is determined by a complex interaction between multiple genes and environmental factors. Thanks to the advent of genome-wide association studies, more than 80 novel genes associated with T1D were identified^7,50^, but their role in the development of T1D remains to be clarified^13,16,64,65^. A large number of T1D candidate genes products are involved in the regulation of the JAK/STAT (e.g. MDA5^51^, TYK2^28^, IRF4^52^, PTPN22^53,54^), and the MAPKs pathways (e.g. NOTCH2^55^, FASLG^56^, TNFAIP3^57^, or NRP1^58^), pointing towards their essential role in the disease and consequently critical “candidate pathways” for future T1D interventions. According to this, treatments targeting the JAK/STAT pathway^21^ and the MAPKs pathway (via TNF inhibition) have shown promising results for the preservation of beta cell function in T1D^19,20^. We presently characterized two of these T1D candidate genes, i.e. *PTPN2* and *BACH2*, showing that they are essential regulators of both JAK/STAT and MAPKs pathways. These observations add evidence to the critical role of these pathways for the beta cell failure in T1D and suggest that a combination of strategies focused on modulating both pathways could be more efficient than targeting them individually. T1D shares several candidate genes in common to other autoimmune diseases such as rheumatoid arthritis, multiple sclerosis, lupus or Crohn’s disease ^9,23,59–61^ and the target tissues of several of these diseases, such as rheumatoid arthritis and lupus, show a similar IFN signature as observed in T1D^4,61^. It is thus conceivable that the present findings may help to understand mechanisms of tissue damage in other diseases where IFNs play a role.^75–77^

It was a surprising finding the presently observed inability of the JAK or TYK2 inhibitors, acting upstream in the JAK/STAT pathway, to completely abolish IFN-α signalling in human beta cells as illustrated by the remaining STAT1-induced expression. The JAK1/2 inhibitor Baricitinib can only block part of the MAPKs pathway through p38 MAPK inhibition, while the TYK2 inhibitor does it through JNK1 inhibition^62^. This suggests that IFN-α responses are more malleable than we initially thought and indicate that these agents may have complementary effects.

In conclusion, in the present study we clarify the complex modulation of IFN-α-mediated STAT1 activation in human beta cells through the crosstalk of two independent pathways, i.e. the JAK/STAT and the MAPKs pathways, and indicate that these pathways can be chemically dissociated. We also show that two T1D candidate genes directly regulate these pathways, providing a link between a “candidate gene pathway” and the downstream molecular mechanisms that contribute to beta cell dysfunction and death in T1D. These findings will be useful to develop novel, and hopefully targeted (i.e. favouring individuals with particular polymorphisms), therapies for T1D and for other autoimmune and inflammatory/degenerative diseases where IFNs play a role.

## Methods

### Culture of human EndoC-βH1, human pancreatic islets and Hela cells

The human pancreatic beta cell line EndoC-βH1 was kindly provided by R Scharfmann (Cochin Institute, France)^63^. Cells were cultured in Matrigel–fibronectin-coated plates as previously described^64^. EndoC-βH1 cells were free of mycoplasma infection, as determined by monthly testing using the MycoAlert Mycoplasma Detection kit (Lonza, Basel, Switzerland).

Human pancreatic islets from 6 non-diabetic organ donors were isolated at the UCL, Brussels, Belgium or at the CHU Lille, France, following a previously described protocol ^65,66^. with written consent from donors’ next-of-kin and approval of the local ethics committee. Information on the organ donors and the isolated islets is provided in Supplementary Table 1.

The human STAT1 knockout (KO) HeLa cell line and its wild-type form were purchased from Abcam (Abcam, Cambridge, UK; ab255346). HeLa cells were grown in 10 ml DMEM/High Glucose supplemented with 10% FBS and 2% penicillin–streptomycin (Thermo Fisher Scientific, Waltham, MA, USA) at 37°C and 5% CO2.

### Differentiation of induced pluripotent stem cells (iPSC) into islet-like cells

The human iPSC line 1023A was kindly provided by DM Egli (Columbia University, NY, USA). The differentiation of iPSCs into islet-like cells was approved by the Ethics Committee of the Erasmus Hospital, Université Libre de Bruxelles, reference P2019/498. iPSCs were cultured in Matrigel-coated plates (Corning, NY, USA) in E8 medium (Invitrogen Life Technologies, Paisley, UK) and passaged with 0.5 mmol/l EDTA (Invitrogen Life Technologies) twice per week. Cell quality and pluripotency were monitored using the MycoAlert Mycoplasma Detection kit for mycoplasma infection, cell karyotyping (Bio.be, Brussels, Belgium) for chromosomal abnormalities and immunocytochemical staining for pluripotency markers as previously described^67^. For beta cell differentiation we used a seven-step protocol previously published by our group^68,69^. Once the differentiation was completed, cell aggregates were dispersed, seeded on Matrigel-coated culture plates and cultured in HAM’s F-10 medium (Thermo Fisher Scientific) containing 2% fatty acid-free BSA (Roche, Basel, Switzerland), 2 mmol/l GlutaMAX (Thermo Fisher Scientific), 50 μM IBMX, and 100 U/ml penicillin–streptomycin (Thermo Fisher Scientific) for exposure to cytokines and/or siRNA as described^67^.

### RNA interference

HeLa cells, EndoC-βH1 cells, or dispersed iPSC-derived islet-like cells were transfected overnight with 30 nmol/l siRNA; the medium was changed, and cells were left to recover for 48 h. Transfection was performed using previously validated siRNAs targeting PTPN2 (siPTPN2; 5′-CACAAAGGAGTTACATCTTAA-3′; 1027415; Qiagen, Venlo, the Netherlands)^17^, BACH2 (siBACH2; 5’-GAUAUUCUCUGUGACGUGATT-3’; S34070, Ambion, Life Technologies Corporation, CA, USA)^14^, JNK1 (also known as MAPK8; siJNK1; 5 ′-GGGCCUACAGAGAGCUAGUUCUUAU-3′; MAPK8HSS108547, Thermo Fisher Scientific)^70^, and p38 MAPK (also known as MAPK14; sip38; 5′-GGAAUUCAAUGAUGUGUAUTT-3′; S1312; Ambion) (validated in this study), using Lipofectamine RNAiMax (Invitrogen) as described^8^. Allstars Negative Control siRNA (siCTRL; Qiagen) was used as a negative control.

### Plasmid transfection

HeLa cells were transfected overnight with 0.5–1 μg/ml plasmid; the medium was changed, and cells were left to recover for 48 h to 72 h. Transfection was performed using Lipofectamine 3000 (Thermo Fisher Scientific) according to the manufacturer’s instructions. pLV-Y701F-STAT1 was a kind gift from George Stark (Addgene plasmid # 71453; http://n2t.net/addgene:71453; RRID: Addgene_71453)^37^. Stat1 alpha S727A pRc/CMV was a kind gift from Jim Darnell (Addgene plasmid # 8700; http://n2t.net/addgene:8700; RRID: Addgene_8700)^36^.

### Exposure to cytokines and chemical drugs

After 24 h to 48 h recovery from silencing, cells were left untreated (NT) or treated with IFN-α (2000 U/ml; Peprotech, London, UK), or TNF-α (1000 U/ml; Peprotech); the specific time-points used are described in the figure legends. The cytokine concentrations were selected based on previous studies from our group^17,71^. C-Jun N-terminal kinase (JNK) was chemically inhibited using SP600125 (Selleck Chemicals, Cologne, Germany). SP600125 optimal concentration to inhibit JNK1 in EndoC-βH1 cells and iPSC-derived islet-like cells, i.e. 20 μmol/l, was determined previously^17^. To disrupt up-stream IFN-α signaling, the JAK1/JAK2 inhibitor Baricitinib (Selleck Chemicals) or the TYK2 inhibitor BMS-986165 (Selleck Chemicals) were used in concentrations previously established by our group^28,72^ (0.4 and 4 μM for Baricitinib; 0.03 and 0.3 μM for BMS-986165). Before the cytokine treatments, cells were pre-treated or not with the specific chemical inhibitor for 2h and them exposed to IFN-α in the continued presence of the inhibitor for the duration described in the figure legends.

### Immunocytochemistry

EndoC-βH1 cells and Hela cells were fixed in 4% paraformaldehyde for 15–20 min, permeabilised with 0.5% triton-X100 in PBS, blocked with UltraV block (Thermo Fisher Scientific) for 10 min and then incubated with primary antibodies diluted in 0.1% Tween in PBS overnight at 4°C. Cells were then washed with PBS and incubated with secondary antibodies diluted in 0.1% Tween in PBS. Samples were mounted with Vectashield with DAPI (Vector Laboratories, Newark, CA, USA) and covered with glass coverslips. The antibodies used in the study are listed in Supplementary Table 2. Images were acquired by widefield fluorescence microscopy (Zeiss, Oberkochen, Germany).

### Nuclei/Cytosol fractionation

Cells were resuspended in 1 ml sucrose solution containing 20 mM Tris pH 7.5–8.0, 100 mM NaCl, 300 mM sucrose, 3 mM MgCl2, and protease inhibitors (Roche) and incubated for 10 min. Next, the solution was centrifuged to pellet nuclei (1000 rcf, 10 min). The supernatant (cytoplasmic fraction) was re-centrifuged (20,000 rcf, 5 min) to pellet debris, and stored at −80°C. Nuclei were then lysed in a high-salt solution containing 20 mM Tris pH 8.0, 300 mM NaCl, and 2 mM EDTA pH 8.0 for 30 minutes, and centrifuged at 20.000 rcf (20 minutes, 4°C). All preparations were performed on ice. The supernatant (nuclei fraction) was stored at −80°C.

### Cell death assay

Cell death was detected by fluorescence microscopy after staining with the DNA binding dyes Hoechst 33342 (5 μg/ml, Sigma Aldrich) and propidium iodide (5 μg/ml, Sigma Aldrich)^17,73^. Cell death was determined in at least 500 cells by two observers, one of them unaware of the experimental conditions.

### Immunoblot

Total protein was extracted using Laemmli or RIPA buffer supplemented with phosphatase and protease inhibitors (Roche) and separated on 10% SDS–PAGE. The nitrocellulose membranes were probed using specific primary antibodies diluted 1:1000 in TBST (TBS, 0.1% Tween 20) with 5% BSA. After overnight incubation at 4°C, membranes were probed for 1 h at room temperature with peroxidase-conjugated secondary antibodies diluted 1:5000 in TBST with 5% BSA. Stripping by low pH (25 mM glycine-HCl, pH 2.2) and reprobing the same membrane to detect different proteins were performed in experiments where the total form of the protein was used to normalize its phosphorylated form. Detection of immunoreactive bands was performed using a chemiluminescent substrate (SuperSignal West Femto, Thermo Fisher Scientific) using a Bio-Rad ChemiDoc XRS + system (Bio-Rad Laboratories, Lokeren, Belgium), or a FUSION FX system (Vilber, Marne-la-Vallée, France). The densitometric values were quantified by ImageJ^74^ and normalised to GAPDH, tubulin or the respective total protein forms, after background subtraction. Antibodies are listed in Supplementary Table 2.

### Real-time PCR and ELISA

Poly(A) + mRNA was isolated using the Dynabeads mRNA DIRECT kit (Invitrogen) according to the manufacturer’s instructions. mRNA molecules were recovered in Tris–HCl elution solution and reverse transcription was performed using the Reverse Transcriptase Core kit (Eurogentec, Liège, Belgium) according to the manufacturer’s instructions. The quantitative reverse transcription PCR (qRTPCR) amplification was conducted using IQ SYBR Green Supermix (Bio-Rad Laboratories). The PCR product concentration was calculated as copies per μl using the standard curve method^75^ and gene expression was normalised to the geometric mean of the reference genes *ACTB* and *VAPA* for human beta cell lines^76^, and to *ACTB* for HeLa cells. Primers are listed in Supplementary Table 3.

The CXCL10 release to the supernatant after 24h or 48h exposure to IFN-α (by 30,000 or 50,000 cells/200 μl) was determined by enzyme-linked immunosorbent assay (Quantikine ELISA kit, R&D Systems, Minneapolis, MN, USA).

### Transcription factor target gene prediction and correlation analysis using the TFTF R package

To predict the target genes of BACH2, we utilized the TFTF R package (v0.1.0), which integrates human transcription factor (TF)-target gene interactions from two major databases: hTFtarget and JASPAR, alongside gene expression data from GTEx (Genotype-Tissue Expression) and TCGA (The Cancer Genome Atlas) for correlation analysis. First, target genes for BACH2 were predicted using the predict_target function from the TFTF package, which employs both hTFtarget and JASPAR databases. The gene expression data from GTEx and TCGA were then used to analyze the correlation between BACH2 expression and its predicted target genes using the pantissue_cor_analysis function. This function performs Pearson correlation analysis, with a focus on pancreatic tissues (both tumor and normal) in our analysis.

### Statistics

Data were analyzed by unpaired t-test, one-way or two-way ANOVA (corrected for repeated measures if required) followed by Bonferroni multiple comparisons tests as required, using GraphPad Prism 8 software (CA, USA). Results are presented as mean ± SEM. p < 0.05 was considered statistically significant. In each experiment, n = 1 is considered to correspond to one independent biological observation, i.e. EndoC-βH1 or HeLa cells from different passages, iPSC-derived islet-like cells from different differentiations, or human islets from different donors. To reduce variability between independent experiments, for some techniques, e.g. qRT-PCR or immunoblot, each independent experiment was normalised against its respective control as specified in the figure legends.

## References

[1] Mathieu C., Martens P. J. & Vangoitsenhoven R. One hundred years of insulin therapy. Nature Reviews Endocrinology 2021 17:12 17, 715–725 (2021). 10.1038/s41574-021-00542-w 34404937

[2] Eizirik D. L., Pasquali L. & Cnop M. Pancreatic β-cells in type 1 and type 2 diabetes mellitus: different pathways to failure. Nature Reviews Endocrinology 2020 16:7 16, 349–362 (2020). 10.1038/s41574-020-0355-7 32398822

[3] Atkinson M. A., Eisenbarth G. S. & Michels A. W. Type 1 diabetes. The Lancet 383, 69–82 (2014). 10.1016/S0140-6736(13)60591-7 PMC4380133 23890997

[4] Coomans de Brachène A. Interferons are key cytokines acting on pancreatic islets in type 1 diabetes. Diabetologia 67, 908–927 (2024). 38409439 10.1007/s00125-024-06106-7

[5] Leete P. The Effect of Age on the Progression and Severity of Type 1 Diabetes: Potential Effects on Disease Mechanisms. Curr Diab Rep 18, 115 (2018). 30259209 10.1007/s11892-018-1083-4 PMC10043737

[6] Tai N., Wong F. S. & Wen L. The role of the innate immune system in destruction of pancreatic beta cells in NOD mice and humans with type I diabetes. J Autoimmun 71, 26–34 (2016). 27021275 10.1016/j.jaut.2016.03.006 PMC4903935

[7] Robertson C. C. Fine-mapping, trans-ancestral and genomic analyses identify causal variants, cells, genes and drug targets for type 1 diabetes. Nature Genetics 2021 53:7 53, 962–971 (2021). 10.1038/s41588-021-00880-5 PMC8273124 34127860

[8] Moore F. PTPN2, a Candidate Gene for Type 1 Diabetes, Modulates Interferon-γ–Induced Pancreatic β-Cell Apoptosis. Diabetes 58, 1283 (2009). 19336676 10.2337/db08-1510 PMC2682688

[9] Sharp R. C., Abdulrahim M., Naser E. S. & Naser S. A. Genetic Variations of PTPN2 and PTPN22: Role in the Pathogenesis of Type 1 Diabetes and Crohn’s Disease. Front Cell Infect Microbiol 5, 95 (2015). 26734582 10.3389/fcimb.2015.00095 PMC4689782

[10] Tiganis T. PTP1B and TCPTP – nonredundant phosphatases in insulin signaling and glucose homeostasis. FEBS J 280, 445–458 (2013). 22404968 10.1111/j.1742-4658.2012.08563.x

[11] Espino-Paisan L. A polymorphism in PTPN2 gene is associated with an earlier onset of type 1 diabetes. Immunogenetics 63, 255–258 (2011). 21246196 10.1007/s00251-010-0500-x

[12] Størling J. & Pociot F. Type 1 Diabetes Candidate Genes Linked to Pancreatic Islet Cell Inflammation and Beta-Cell Apoptosis. Genes (Basel) 8, (2017). 10.3390/genes8020072 PMC5333061 28212332

[13] Miele L. Genetic susceptibility of increased intestinal permeability is associated with progressive liver disease and diabetes in patients with non-alcoholic fatty liver disease. Nutrition, Metabolism and Cardiovascular Diseases 30, 2103–2110 (2020). 10.1016/j.numecd.2020.06.013 32807638

[14] Marroquí L. BACH2, a candidate risk gene for type 1 diabetes, regulates apoptosis in pancreatic β-cells via JNK1 modulation and crosstalk with the candidate gene PTPN2. Diabetes 63, 2516–2527 (2014). 24608439 10.2337/db13-1443

[15] Son J. BACH2 inhibition reverses β cell failure in type 2 diabetes models. J Clin Invest 131, (2021). 10.1172/JCI153876 PMC8670842 34907913

[16] Roychoudhuri R. The transcription factor BACH2 promotes tumor immunosuppression. J Clin Invest 126, 599–604 (2016). 26731475 10.1172/JCI82884 PMC4731158

[17] Roca-Rivada A. Inhibition of the type 1 diabetes candidate gene PTPN2 aggravates TNF-α-induced human beta cell dysfunction and death. Diabetologia 66, 1544–1556 (2023). 36988639 10.1007/s00125-023-05908-5

[18] Achenbach P. A classification and regression tree analysis identifies subgroups of childhood type 1 diabetes. EBioMedicine 82, 104118 (2022). 35803018 10.1016/j.ebiom.2022.104118 PMC9270253

[19] Quattrin T. Golimumab and Beta-Cell Function in Youth with New-Onset Type 1 Diabetes. New England Journal of Medicine 383, 2007–2017 (2020). 33207093 10.1056/NEJMoa2006136

[20] Rigby M. R. Two-Year Follow-up From the T1GER Study: Continued Off-Therapy Metabolic Improvements in Children and Young Adults With New-Onset T1D Treated With Golimumab and Characterization of Responders. Diabetes Care 46, 561–569 (2023). 36576974 10.2337/dc22-0908 PMC10020023

[21] Waibel M. Baricitinib and β-Cell Function in Patients with New-Onset Type 1 Diabetes. N Engl J Med 389, 2140–2150 (2023). 38055252 10.1056/NEJMoa2306691

[22] Hoffmann P., Lamerz D., Hill P., Kirchner M. & Gauss A. Gene Polymorphisms of NOD2, IL23R, PTPN2 and ATG16L1 in Patients with Crohn’s Disease: On the Way to Personalized Medicine? Genes (Basel) 12, (2021). 10.3390/genes12060866 PMC8227795 34198814

[23] Sharp R. C., Beg S. A. & Naser S. A. Polymorphisms in Protein Tyrosine Phosphatase Non-receptor Type 2 and 22 (PTPN2/22) Are Linked to Hyper-Proliferative T-Cells and Susceptibility to Mycobacteria in Rheumatoid Arthritis. Front Cell Infect Microbiol 8, (2018). 10.3389/fcimb.2018.00011 PMC5788942 29423382

[24] McAllister K. Identification of BACH2 and RAD51B as rheumatoid arthritis susceptibility loci in a meta-analysis of genome-wide data. Arthritis Rheum 65, 3058–3062 (2013). 24022229 10.1002/art.38183 PMC4034583

[25] Moore F. STAT1 Is a Master Regulator of Pancreatic β-Cell Apoptosis and Islet Inflammation. J Biol Chem 286, 929 (2011). 20980260 10.1074/jbc.M110.162131 PMC3020778

[26] Sidarala V. & Kowluru A. The Regulatory Roles of Mitogen-Activated Protein Kinase (MAPK) Pathways in Health and Diabetes: Lessons Learned from the Pancreatic β-Cell. Recent Pat Endocr Metab Immune Drug Discov 10, 76 (2017). 27779078 10.2174/1872214810666161020154905 PMC6685434

[27] Ortis F. Differential usage of NF-κB activating signals by IL-1β and TNF-α in pancreatic beta cells. FEBS Lett 586, 984–989 (2012). 22569251 10.1016/j.febslet.2012.02.021

[28] Coomans de Brachène A. Preclinical evaluation of tyrosine kinase 2 inhibitors for human beta-cell protection in type 1 diabetes. Diabetes Obes Metab 22, 1827 (2020). 32476252 10.1111/dom.14104 PMC8080968

[29] Santin I. PTPN2, a candidate gene for type 1 diabetes, modulates pancreatic β-cell apoptosis via regulation of the BH3-only protein bim. Diabetes 60, 3279–3288 (2011). 21984578 10.2337/db11-0758 PMC3219938

[30] Song J. H., So E. Y. & Lee C. E. Increased Serine Phosphorylation and Activation of STAT1 by Oncogenic Ras Transfection. Mol Cells 13, 322–326 (2002). 12018856

[31] Chodisetti S. B. Serine phosphorylation of the STAT1 transactivation domain promotes autoreactive B cell and systemic autoimmunity development. J Immunol 204, 2641 (2020). 32253245 10.4049/jimmunol.2000170 PMC9305983

[32] Roychoudhuri R. BACH2 regulates CD8+ T cell differentiation by controlling access of AP-1 factors to enhancers. Nature Immunology 2016 17:7 17, 851–860 (2016). 10.1038/ni.3441 PMC4918801 27158840

[33] Xu N. MicroRNA-31 Is Overexpressed in Psoriasis and Modulates Inflammatory Cytokine and Chemokine Production in Keratinocytes via Targeting Serine/Threonine Kinase 40. The Journal of Immunology 190, 678–688 (2013). 23233723 10.4049/jimmunol.1202695

[34] Bunz M. CD81 suppresses NF-κB signaling and is downregulated in hepatitis C virus expressing cells. Front Cell Infect Microbiol 14, (2024). 10.3389/fcimb.2024.1338606 PMC10864554 38357447

[35] Liu T., Zhang L., Joo D. & Sun S. C. NF-κB signaling in inflammation. Signal Transduction and Targeted Therapy 2017 2:1 2, 1–9 (2017). 10.1038/sigtrans.2017.23 PMC5661633 29158945

[36] Wen Z., Zhong Z. & Darnell J. E. Maximal activation of transcription by Stat1 and Stat3 requires both tyrosine and serine phosphorylation. Cell 82, 241–250 (1995). 7543024 10.1016/0092-8674(95)90311-9

[37] Cheon H. J. & Stark G. R. Unphosphorylated STAT1 prolongs the expression of interferon-induced immune regulatory genes. Proc Natl Acad Sci U S A 106, 9373–9378 (2009). 19478064 10.1073/pnas.0903487106 PMC2688000

[38] Colli M. L. PDL1 is expressed in the islets of people with type 1 diabetes and is up-regulated by interferons-α and-γ via IRF1 induction. EBioMedicine 36, 367–375 (2018). 30269996 10.1016/j.ebiom.2018.09.040 PMC6197434

[39] Goh K. C., Haque S. J. & Williams B. R. G. p38 MAP kinase is required for STAT1 serine phosphorylation and transcriptional activation induced by interferons. EMBO J 18, 5601 (1999). 10523304 10.1093/emboj/18.20.5601 PMC1171628

[40] Zhang Y., Cho Y. Y., Petersen B. L., Zhu F. & Dong Z. Evidence of STAT1 phosphorylation modulated by MAPKs, MEK1 and MSK1. Carcinogenesis 25, 1165–1175 (2004). 14963018 10.1093/carcin/bgh115

[41] Scharl M., Hruz P. & McCole D. F. Protein tyrosine phosphatase non-receptor Type 2 regulates IFN-γ-induced cytokine signaling in THP-1 monocytes. Inflamm Bowel Dis 16, 2055–2064 (2010). 20848498 10.1002/ibd.21325

[42] Tolomeo M., Cavalli A. & Cascio A. STAT1 and Its Crucial Role in the Control of Viral Infections. Int J Mol Sci 23, 4095 (2022). 35456913 10.3390/ijms23084095 PMC9028532

[43] Metwally H. Threonine phosphorylation of STAT1 restricts interferon signaling and promotes innate inflammatory responses. Proc Natl Acad Sci U S A 121, e2402226121 (2024). 38621137 10.1073/pnas.2402226121 PMC11046697

[44] Sadzak I. Recruitment of Stat1 to chromatin is required for interferon-induced serine phosphorylation of Stat1 transactivation domain. Proc Natl Acad Sci U S A 105, 8944–8949 (2008). 18574148 10.1073/pnas.0801794105 PMC2435588

[45] Varinou L. Phosphorylation of the Stat1 transactivation domain is required for full-fledged IFN-gamma-dependent innate immunity. Immunity 19, 793–802 (2003). 14670297 10.1016/s1074-7613(03)00322-4

[46] Kovarik P. Stress-induced phosphorylation of STAT1 at Ser727 requires p38 mitogen-activated protein kinase whereas IFN-gamma uses a different signaling pathway. Proc Natl Acad Sci U S A 96, 13956–13961 (1999). 10570180 10.1073/pnas.96.24.13956 PMC24172

[47] Zhang Y., Cho Y. Y., Petersen B. L., Zhu F. & Dong Z. Evidence of STAT1 phosphorylation modulated by MAPKs, MEK1 and MSK1. Carcinogenesis 25, 1165–1175 (2004). 14963018 10.1093/carcin/bgh115

[48] Crow Y. J. & Stetson D. B. The type I interferonopathies: 10 years on. Nature Reviews Immunology 2021 22:8 22, 471–483 (2021). 10.1038/s41577-021-00633-9 PMC8527296 34671122

[49] Chodisetti S. B. Serine phosphorylation of the STAT1 transactivation domain promotes autoreactive B cell and systemic autoimmunity development. J Immunol 204, 2641 (2020). 32253245 10.4049/jimmunol.2000170 PMC9305983

[50] Bakay M., Pandey R. & Hakonarson H. Genes Involved in Type 1 Diabetes: An Update. Genes (Basel) 4, 499 (2013). 24705215 10.3390/genes4030499 PMC3924830

[51] Li Y. MDA5 against enteric viruses through induction of interferon-like response partially via the JAK-STAT cascade. Antiviral Res 176, (2020). 10.1016/j.antiviral.2020.104743 32057771

[52] Das Gupta D. IRF4 deficiency vulnerates B-cell progeny for leukemogenesis via somatically acquired Jak3 mutations conferring IL-7 hypersensitivity. Cell Death Differ 29, 2163 (2022). 35459909 10.1038/s41418-022-01005-z PMC9613660

[53] Bottini N. & Peterson E. J. Tyrosine phosphatase PTPN22: multifunctional regulator of immune signaling, development, and disease. Annu Rev Immunol 32, 83 (2013). 24364806 10.1146/annurev-immunol-032713-120249 PMC6402334

[54] Fabbri M., Frixou M., Degano M. & Fousteri G. Type 1 Diabetes in STAT Protein Family Mutations: Regulating the Th17/Treg Equilibrium and Beyond. Diabetes 68, 258–265 (2019). 30665954 10.2337/db18-0627

[55] Wang J. Notch2 controls hepatocyte-derived cholangiocarcinoma formation in mice. Oncogene 37, 3229 (2018). 29545603 10.1038/s41388-018-0188-1 PMC6002343

[56] Abreu-Martin M. T. Fas activates the JNK pathway in human colonic epithelial cells: lack of a direct role in apoptosis. Am J Physiol 276, (1999). 10.1152/ajpgi.1999.276.3.G599 10070035

[57] Pinna F. A20/TNFAIP3 Discriminates Tumor Necrosis Factor (TNF)-Induced NF-κB from JNK Pathway Activation in Hepatocytes. Front Physiol 8, (2017). 10.3389/fphys.2017.00610 PMC5572400 28878689

[58] Rizzolio S. Neuropilin-1 upregulation elicits adaptive resistance to oncogene-targeted therapies. J Clin Invest 128, 3976 (2018). 29953416 10.1172/JCI99257 PMC6118581

[59] Yu W. PTPN2 is associated with Crohn’s disease and its expression is regulated by NKX2–3. Dis Markers 32, 83–91 (2012). 22377701 10.3233/DMA-2011-0867 PMC3826479

[60] Conigliaro P. Polymorphisms in STAT4, PTPN2, PSORS1C1 and TRAF3IP2 Genes Are Associated with the Response to TNF Inhibitors in Patients with Rheumatoid Arthritis. PLoS One 12, (2017). 10.1371/journal.pone.0169956 PMC5249113 28107378

[61] Szymczak F., Colli M. L., Mamula M. J., Evans-Molina C. & Eizirik D. L. Gene expression signatures of target tissues in type 1 diabetes, lupus erythematosus, multiple sclerosis, and rheumatoid arthritis. Sci Adv 7, (2021). 10.1126/sciadv.abd7600 PMC7787485 33523973

[62] Mazewski C., Perez R. E., Fish E. N. & Platanias L. C. Type I Interferon (IFN)-Regulated Activation of Canonical and Non-Canonical Signaling Pathways. Front Immunol 11, (2020). 10.3389/fimmu.2020.606456 PMC7719805 33329603

[63] Ravassard P. A genetically engineered human pancreatic β cell line exhibiting glucose-inducible insulin secretion. J Clin Invest 121, 3589–3597 (2011). 21865645 10.1172/JCI58447 PMC3163974

[64] Brozzi F. Cytokines induce endoplasmic reticulum stress in human, rat and mouse beta cells via different mechanisms. Diabetologia 58, 2307–2316 (2015). 26099855 10.1007/s00125-015-3669-6

[65] Marroqui L. Differential cell autonomous responses determine the outcome of coxsackievirus infections in murine pancreatic α and β cells. Elife 4, 1–23 (2015). 10.7554/eLife.06990 PMC4480275 26061776

[66] Marchetti P., Suleiman M. & Marselli L. Organ donor pancreases for the study of human islet cell histology and pathophysiology: a precious and valuable resource. Diabetologia 61, 770–774 (2018). 29354869 10.1007/s00125-018-4546-x PMC6449064

[67] Lytrivi M. DNAJC3 deficiency induces β-cell mitochondrial apoptosis and causes syndromic young-onset diabetes. Eur J Endocrinol 184, 455–468 (2021). 33486469 10.1530/EJE-20-0636

[68] Cosentino C. Pancreatic β-cell tRNA hypomethylation and fragmentation link TRMT10A deficiency with diabetes. Nucleic Acids Res 46, 10302–10318 (2018). 30247717 10.1093/nar/gky839 PMC6212784

[69] Fantuzzi F. In depth functional characterization of human induced pluripotent stem cell-derived beta cells in vitro and in vivo. Front Cell Dev Biol 10, (2022). 10.3389/fcell.2022.967765 PMC9428245 36060810

[70] Dos Santos R. S. Protective Role of Complement C3 Against Cytokine-Mediated β-Cell Apoptosis. Endocrinology 158, 2503 (2017). 28582497 10.1210/en.2017-00104 PMC5551554

[71] Szymczak F. Transcription and splicing regulation by NLRC5 shape the interferon response in human pancreatic β cells. Sci Adv 8, (2022). 10.1126/sciadv.abn5732 PMC9473574 36103539

[72] Colli M. L. An integrated multi-omics approach identifies the landscape of interferon-α-mediated responses of human pancreatic beta cells. Nat Commun 11, (2020). 10.1038/s41467-020-16327-0 PMC7244579 32444635

[73] Hoorens A., Van De Casteele M., Klöppel G. & Pipeleers D. Glucose Promotes Survival of Rat Pancreatic Cells by Activating Synthesis of Proteins Which Suppress a Constitutive Apoptotic Program. J. Clin. Invest 98, 1568–1574 (1996). 8833905 10.1172/JCI118950 PMC507589

[74] Schneider C. A., Rasband W. S. & Eliceiri K. W. NIH Image to ImageJ: 25 years of image analysis. Nature Methods 2012 9:7 9, 671–675 (2012). 10.1038/nmeth.2089 PMC5554542 22930834

[75] Overbergh L., Valckx D., Waer M. & Mathieu C. Quantification of murine cytokine mRNAs using real time quantitative reverse transcriptase PCR. Cytokine 11, 305–312 (1999). 10328870 10.1006/cyto.1998.0426

[76] Alvelos M. I. A functional genomic approach to identify reference genes for human pancreatic beta cell real-time quantitative RT-PCR analysis. Islets 13, 51–65 (2021). 34241569 10.1080/19382014.2021.1948282 PMC8280887

